# Interplay between metabolic reprogramming and post-translational modifications: from glycolysis to lactylation

**DOI:** 10.3389/fimmu.2023.1211221

**Published:** 2023-06-29

**Authors:** Hengwei Wu, He Huang, Yanmin Zhao

**Affiliations:** ^1^ Bone Marrow Transplantation Center, The First Affiliated Hospital, Zhejiang University School of Medicine, Hangzhou, Zhejiang, China; ^2^ Institute of Hematology, Zhejiang University, Hangzhou, Zhejiang, China; ^3^ Zhejiang Province Engineering Laboratory for Stem Cell and Immunity Therapy, People's Government of Zhejiang Province, Hangzhou, Zhejiang, China; ^4^ Zhejiang Laboratory for Systems & Precision Medicine, Zhejiang University Medical Center, Hangzhou, Zhejiang, China

**Keywords:** metabolic reprogramming, epigenetics, glycolysis, lactate, lactylation

## Abstract

Cellular metabolism plays a critical role in determining the fate and function of cells. Metabolic reprogramming and its byproducts have a complex impact on cellular activities. In quiescent T cells, oxidative phosphorylation (OXPHOS) is the primary pathway for survival. However, upon antigen activation, T cells undergo rapid metabolic reprogramming, characterized by an elevation in both glycolysis and OXPHOS. While both pathways are induced, the balance predominantly shifts towards glycolysis, enabling T cells to rapidly proliferate and enhance their functionality, representing the most distinctive signature during activation. Metabolic processes generate various small molecules resulting from enzyme-catalyzed reactions, which also modulate protein function and exert regulatory control. Notably, recent studies have revealed the direct modification of histones, known as lactylation, by lactate derived from glycolysis. This lactylation process influences gene transcription and adds a novel variable to the regulation of gene expression. Protein lactylation has been identified as an essential mechanism by which lactate exerts its diverse functions, contributing to crucial biological processes such as uterine remodeling, tumor proliferation, neural system regulation, and metabolic regulation. This review focuses on the metabolic reprogramming of T cells, explores the interplay between lactate and the immune system, highlights the impact of lactylation on cellular function, and elucidates the intersection of metabolic reprogramming and epigenetics.

## 1 Introduction

The Nobel Prize in Physiology or Medicine 2019 was jointly awarded to three scientists for their discovery of how cells sense and adjust to oxygen levels. Oxygen sensing and energy metabolism play vital roles in numerous physiological and pathological processes. Tumors exhibit a unique energy metabolism known as the Warburg effect, where they preferentially use glycolysis to produce energy and accumulate substantial amounts of lactate, even under aerobic conditions. The Warburg effect is not exclusive to tumors but also occurs in several other diseases, such as sepsis, autoimmune disorders, atherosclerosis, diabetes, and ageing. Quiescent T cells primarily utilize oxidative phosphorylation (OXPHOS) for energy supply. However, upon antigen activation, these cells undergo rapid metabolic reprogramming and switch to aerobic glycolysis. This shift in metabolism supports T cell proliferation and enhances their functionality, representing a significant metabolic change during activation (1). For a long time, the accumulated lactate produced from glycolysis was viewed as a mere cellular energy source and metabolic byproduct, with its critical regulatory function in biological processes receiving inadequate attention. Lactate acts not only as a significant signaling molecule but also as a substrate for post-translational protein modification (2). As a ubiquitous metabolite, lactate still requires comprehensive elucidation of its role in physiological and pathological processes through post-translational modifications. This review will center on the critical biological process of metabolic reprogramming in immune cells, followed by an investigation of lactate’s effects on cellular activity

## 2 Metabolic reprogramming is vital for immune activities

Metabolic reprogramming is an adaptive modulation of cellular energy requirements to enable new functions in response to distinct environmental conditions. The Warburg effect is the archetypical example of metabolic reprogramming, wherein proliferating tumor cells metabolize glucose at a heightened rate *via* glycolysis, even under aerobic conditions, ultimately producing lactate. This phenomenon is widespread in many types of tumors (3, 4). Macrophages exhibit high plasticity and their polarization is influenced by stimuli from their microenvironment, which drives them to acquire distinct functions. Typically, macrophages are categorized into M1 (pro-inflammatory) and M2 (anti-inflammatory) subsets based on their function. The M2 macrophage subset exhibits activation of OXPHOS and fatty acid oxidation (FAO), whereas M1 macrophages utilize aerobic glycolysis (2). The metabolic pathways of pro-inflammatory cells, such as CD4^+^ effector T cells, are primarily shaped by glycolysis. In contrast, anti-inflammatory T cells, such as memory CD8^+^ effector T cells and Treg cells, are typically supported by OXPHOS and FAO. This section highlights the critical significance of metabolic reprogramming by focusing on T cells.

### 2.1 Elevated glycolysis is indispensable for eliciting active and functional T cells

When T cells are activated through CD3/CD28 *in vitro*, they undergo division within the following 24 to 72 hours. Thus, the first 24 hours are considered a “window” to determine the metabolic reprogramming profile upon activation (5). During rapid differentiation and proliferation, T cells upregulate both OXPHOS and glycolysis (6). Of note, the increase in OXPHOS is transient and serves as a characteristic of early activated T cells (7). Therefore, there is a dramatic shift in glucose utilization towards aerobic glycolysis, which facilitates the activation of the pentose phosphate pathway (PPP) and increased consumption of glutamine. This metabolic transition has significant implications, such as retaining some extent of oxygen molecules for other vital cellular processes that require oxygen during hypoxia and producing metabolic intermediates necessary for maintaining cellular activity and generating daughter cells (8, 9). This close connection between T cell activation and metabolism suggests that changes in T cell metabolism are not just a result of activation, but a factor that influences T cell fate. For instance, CD4^+^ effector memory T cells rely on glycolysis to avoid apoptosis (10).

**Figure 1:**
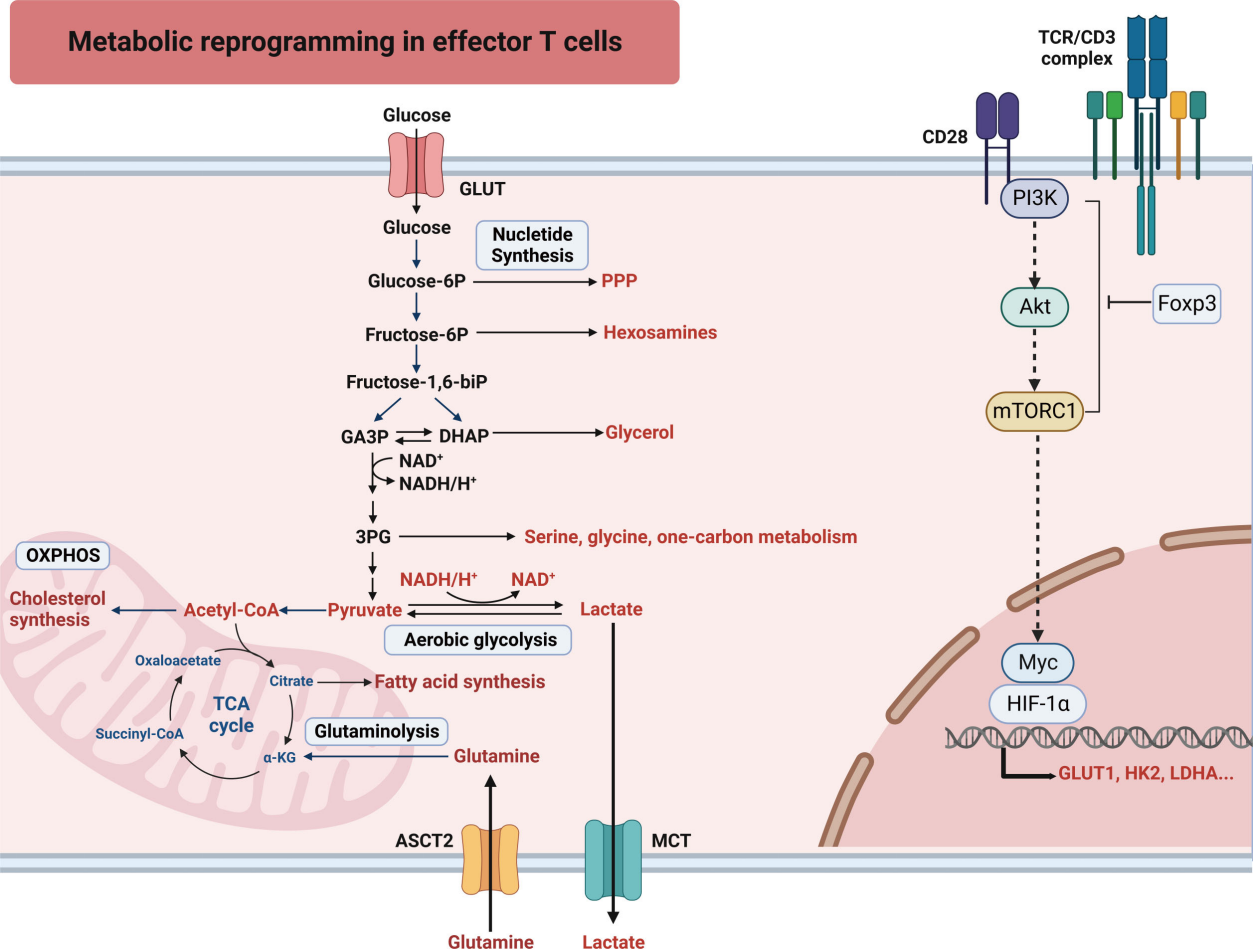
T cells undergo an increased uptake of glucose and glutamine to support the metabolic shift characterized by glycolysis, glutaminolysis and PPP. To sustain heightened glycolytic activity and ATP synthesis, prompt restoration of NAD^+^ from NADH is imperative. Hence, activated T cells facilitate this process by efficiently converting pyruvate, the final product of glycolysis, into lactate. 3-PG, 3-phosphoglyceric acid; HIF-1α, hypoxia-induced factor 1 alpha; Akt, acetyl coenzyme A; ASCT2, alanine serine cysteine transporter 1; Foxp3, forkhead box protein p3; GLUT, glucose transporter; HK2, hexokinase 2; LDHA, lactate dehydrogenase A; MCT, monocarboxylate transporter; mTORC1, mammalian target of rapamycin complex 1; OXPHOS, oxidative phosphorylation; PI3K, phosphoinositide 3-kinases; PPP, pentose phosphate pathway; SLC, solute carrier transporter; TCA cycle, tricarboxylic acid cycle; TCR, T cell receptor.

Active aerobic glycolysis is a lineage-decisive step for T cells. The activation of the phosphoinositide 3-kinases (PI3K)/acetyl coenzyme A (Akt)/mammalian target of rapamycin (mTOR) pathway or the regulation by transcription factors Myc and hypoxia-induced factor 1 α (HIF-1α) can induce aerobic glycolysis in effector T cells. mTOR is composed of two complexes: mTOR complex 1 (mTORC1) and mTORC2. CD4^+^ T cells that lack mTORC1 cannot differentiate into Th1, Th2, or Th17 lineages (11). Stimulation of the T cell receptor (TCR) activates the PI3K/Akt/mTOR signaling pathway. mTOR activates HIF-1α and Myc to enhance glycolysis, glutaminolysis, and PPP (**Figure 1**) (5). The pathway plays a crucial role in regulating the expression and transport of glucose transporter 1 (GLUT1) (12). HIF-1α and Myc increase the expression of enzymes involved in glycolysis and glutaminolysis, as well as transporters for glucose and glutamine influx (1, 5, 13, 14). It is interesting to note that Myc-mediated glutamine catabolism works in concert with Myc-mediated glucose catabolism (5). This metabolic reprogramming not only aids in the differentiation of T cells towards effector T cells but also facilitates the formation of effector memory T cells (15). The metabolic activity and proliferation of cells create a hypoxic environment that leads to an increase in HIF-1α expression and further boosts glycolysis in low oxygen conditions (13, 16). HIF-1α reduces the differentiation of Th17 cells and enhances the differentiation of suppressive Treg cells (13). The Toll-like receptor (TLR) signals regulate the function of Treg cells. TLR1 and TLR2 promote glycolysis of Treg cells but impair suppression (17). But the deficient TLR adaptor-transducer MyD88 leads to a reduction of Treg cells in the gut, contributing to the development of inflammatory bowel disease (18).

Maintaining glycolysis is crucial for the production of interferon-γ (IFN-γ). The expression of lactate dehydrogenase A (LDHA) is increased after cellular reprogramming to aerobic glycolysis. T cells with a deficiency of LDHA (*Lhda* ^KO^ T cells) have decreased IFN-γ expression, which ameliorates autoimmune diseases without impacting the expression of T-bet (19, 20). LDHA plays a role in maintaining the level of acetyl-CoA to some extent. Acetyl-CoA serves as a substrate for histone acetylation and this process, in turn, promotes the transcription of *Ifng* (20). Glyceraldehyde-3-phosphate dehydrogenase (GAPDH), a key enzyme involved in glycolysis, also binds to the AU-rich region of the 3′ untranslated region of *Ifng* mRNA and hinders its translation (21). The relationships between high glycolysis and inflammation (22), infections (23), and autoimmune diseases (24) are well established. CD4^+^ T cells differentiation into Th1 and Th17 lineages require glycolysis, thus the inhibition of glycolysis has been shown to ameliorate autoimmune encephalitis (25). However, impaired T-cell glycolysis has been observed in some autoimmune diseases associated with T cell dysfunction. Reduced expression of the rate-limiting glycolytic enzyme, 6-phosphofructokinase-2/fructobisphosphatase-2 isoenzyme 3 (PFKFB3), has been observed in CD4^+^ T cells from patients with rheumatoid arthritis. This reduction in PFKFB3 leads to decreased glycolysis, shifting the flow of glucose towards PPP, thereby depleting intracellular reactive oxygen species (ROS). As a result, these T cells exhibit a reduced capacity for energy generation and biosynthesis *via* autophagy (26).

### 2.2 Other metabolic features orchestrate with glycolysis tailoring immune response

T cell subsets exhibit distinct metabolic requirements for proliferation, differentiation, and function (21). While most helper T cells (Th1, Th2, Th17) rely mainly on aerobic glycolysis, Treg cells display heterogeneous metabolic profiles, with a reliance on FAO and OXPHOS as their primary energy sources (27–29). This does not negate the importance of glycolysis in Treg cell biology. Inhibition of Treg cell glycolysis impairs their suppressive activity and decreases Foxp3 expression by recruiting enolase-1 (an enzyme of the glycolytic enzyme pathway) to the Foxp3 motif to control variable splicing (30). In addition, tumor-associated Treg cells exhibit high glucose uptake and glycolysis (31), while Foxp3 suppression of glycolysis through Myc decreases PI3K/Akt/mTORC1 signaling and escalates OXPHOS (17, 32). Many studies have found that if activated T cells are to differentiate into Treg cells, they must restrict their glycolysis levels and undergo a metabolic shift towards OXPHOS (28, 33, 34). Therefore, T cells initially exist in an OXPHOS state, undergo increased glycolysis upon activation, and finally tilt the balance of their metabolic profile towards OXPHOS to generate Treg cells. This highlights the interplay between metabolic processes and their mutual regulation.

T cell activation results in increased expression of genes regulating fatty acid synthesis (FASN) (35). Acetyl-CoA carboxylase (ACC) 1 and ACC2 are rate-limiting enzymes in FASN (11). The development of Th17 cells depends on ACC1-mediated FASN to synthesize phospholipids for cell membranes (36). Inhibition of ACC1 impairs the generation of Th17, Th1, and Th2 cells and leads to the development of Foxp3^+^ Treg cells, which ameliorates experimental autoimmune encephalomyelitis (33). Conversely, intratumoral Treg cells primarily rely on FASN for proliferation and mature Treg cell generation, rather than fatty acids (FAs) uptake (31, 37). Activated Tregs cells exhibit a transcription profile characterized by increased glycolysis and lipid biosynthesis (31). Interestingly, cells require essential FAs from the environment despite utilizing *de novo* FASN (38). Intratumoral Treg cells upregulate fatty acid-binding proteins and CD36, key facilitators of FAs uptake (39, 40). Meanwhile, there is a robust correlation between FAO and long-lived memory T cells (41). It’s interesting to note that memory T cells utilize glucose absorption for FASN and subsequently FAO, instead of utilizing extracellular free fatty acids for FAO (42). Although an initial increase in FAO during T cell activation, inhibition of FAO does not impede the proliferation and function of activated effector T cells (1, 43). Stimulation of T cells by PD-1 signaling leads to a shift in metabolism from glycolysis to fatty acid oxidation, which may contribute to the suppressive effect of PD-1 on T cell function (44).

As aforementioned, T cell activation is coupled with upregulated PPP, but blocking PPP has little effect on CD4^+^ T cell proliferation and activation *in vivo*, suggesting alternative pathways may support these processes (5). Glutaminase (GLS) converts glutamine to glutamate to support the tricarboxylic acid (TCA) cycle and epigenetic regulation. GLS deficiency impairs early T cell activation and reduces Th17 differentiation while increasing T-bet-related Th1 differentiation through altered gene expression and chromatin accessibility (45). These findings highlight the rapid, flexible, and reversible metabolic reprogramming and accompanying epigenetic changes that occur during T cell activation and differentiation.

### 2.3 Implications of immunometabolism on T cell biology

To meet the energy and metabolic demands for rapid proliferation, tumor cells uptake large amounts of glucose for glycolysis, resulting in glucose depletion in the tumor microenvironment (TME) and thereby inhibiting T cell glycolysis and subsequent anti-tumor response (46). In addition to correcting the unfavorable environment for T cells in the TME, it is also possible to enhance their function by targeting T cells themselves. Pre-clinical studies have revealed that the differentiation of CD8^+^ T cells into memory T cells could enhance their anti-tumor (47) and anti-viral capabilities (48), primarily due to improved survival and proliferative capacities. It has been established that CD8^+^ memory T cells primarily rely on FAO for energy production. Deficiencies in FAO fail to generate sufficient numbers of CD8^+^ memory T cells (49), while overexpression of carnitine palmitoyltransferase 1a (a rate-limiting enzyme of FAO) increases the abundance of memory T cells (50). Conversely, a metabolic state characterized by high glycolytic activity hinders the functional development of CD8^+^ memory T cells, driving the cells towards an effector T cell phenotype and subsequent exhaustion; inhibiting the glycolytic metabolism of T cells enhances the formation of memory phenotype and augments their anti-tumor effects (51).

As previously mentioned, T-cell activation leads to an upregulation of glucose transporter proteins, enhancing glucose uptake. Studies have demonstrated that CD4^+^ T cells infected with human immunodeficiency virus 1 (HIV-1) exhibit increased glycolysis and upregulation of GLUT1 expression, directly facilitating viral replication (52). Furthermore, the human T lymphotropic virus even exploits GLUT1 as an entry receptor and upregulates GLUT1 expression to facilitate subsequent infection (53). HIV-1 controllers (HICs) from CD8^+^ T cells represent a rare subset of individuals with a remarkable innate capacity to suppress viral replication without therapeutic interventions (54). HICs exhibit a distinct characteristic of reduced glycolysis and memory phenotype (55). Interestingly, non-HIC CD8^+^ T cells, upon treatment, display heightened fatty acid uptake and enhanced OXPHOS, demonstrating an augmented ability to suppress HIV-1 (55). Studies have shown that inhibiting glycolysis with lactose or 2-DG can significantly suppress HIV-1 infection (56, 57). Hence, analogous to tumorigenesis, restraining T cell glycolysis and enhancing FAO and OXPHOS seem to facilitate the development of memory phenotypes and extend cellular survival, constituting a promising yet nascent field warranting further investigation.

## 3 The glycolytic metabolite lactate is an important signaling molecule that shapes the function of immune cells

Aerobic glycolysis plays a crucial role in providing rapid energy in the form of ATP and producing intermediates for biosynthesis. However, the claim has been debated, as the majority of glucose carbon (more than 90%) is secreted as lactate and alanine, leaving limited room for biosynthesis (58). Creatine kinase and adenylate kinase can also promptly produce ATP (59, 60). Metabolites that are more likely to regulate cellular activities are of particular interest (21, 61). Altered metabolism rate changes the levels of intermediates and metabolites, endowing these small molecules with signaling functions (62). Hence, it is important to explore the potential functional and survival advantages that small molecules from metabolic processes bring to cells.

Studies on the relationship between immune cells and energy metabolism have primarily explored the impact of metabolic pathways on the immune system. Recent findings have revealed metabolites such as lactate, acetyl-CoA, and succinate play a crucial role in immune regulation as signaling molecules. Cellular activity generates a multitude of metabolites with various functions, including the regulation of cytokine production through alterations in cellular redox status, influencing transcriptional processes through binding to cytokine initiators, and modulating transmembrane ion channels, cell migration, and differentiation (63–65). Metabolites, therefore, serve as messengers in cell-cell communication and response to the microenvironment, transcending their original definition as mere metabolic intermediates.

### 3.1 Lactate derived from glycolysis

Lactate, a byproduct of glycolysis, is transported out of the cytoplasm and subsequently deposited in the extracellular space. The conversion of pyruvate to lactate or lactate to pyruvate is catalyzed by lactate dehydrogenases (LDHs), which exist as five tetrameric isoenzymes (A4B0, A3B1, A2B2, A1B3, and A0B4) composed of LDHA and LDHB subunits (66). LDHB has a higher affinity for lactate and converts it to pyruvate, while LDHA favors the reverse reaction. Even though immune cells can express LDHA and LDHB subunits, it is interesting to note that T cells primarily express A4B0, which is increased during activation (20).

Lactate and protons are transported *via* reversible monocarboxylate transporters (MCTs) and their transport direction is determined by the concentration gradients of both monocarboxylate ions and protons. Different MCTs can facilitate intercellular lactic acid transport, while sodium-coupled lactate (e.g., Na-lactate) transport is mediated by solute carrier (SLC) 5A8 or SLC5A12 (67). Notably, CD4^+^ and CD8^+^ T cells detect heightened lactate levels upon infiltration of inflammatory sites through SLC5A12 and MCT1 (SLC16A1) transporters, respectively (64).

Historically, lactate was viewed merely as a waste product of metabolism and a biomarker for critically ill patients, neglecting its role as a bioactive molecule with major impacts on immune cells in the microenvironment. Lactate accumulation in the microenvironment significantly impacts immune cells, both tissue-resident and infiltrating. Lactate accumulated milieu can help evade immunosurveillance by conferring immunosuppressive functions to macrophages and T cells or amplifying inflammatory signals in inflammatory diseases (32, 63, 64, 68, 69). In certain healthy and malignant tissues, lactate utilization even outperforms glucose utilization as an energy source (70, 71). Lactate also acts as a signaling molecule when it binds to G-protein-coupled receptor 81 (GPR81), which is involved in lipolysis (72) and cancer cell survival (73). However, it remains unclear if using lactate as a carbon source also supports its other functions.

### 3.2 Lactate exerts its function *via* a receptor-independent manner

**Figure 2:**
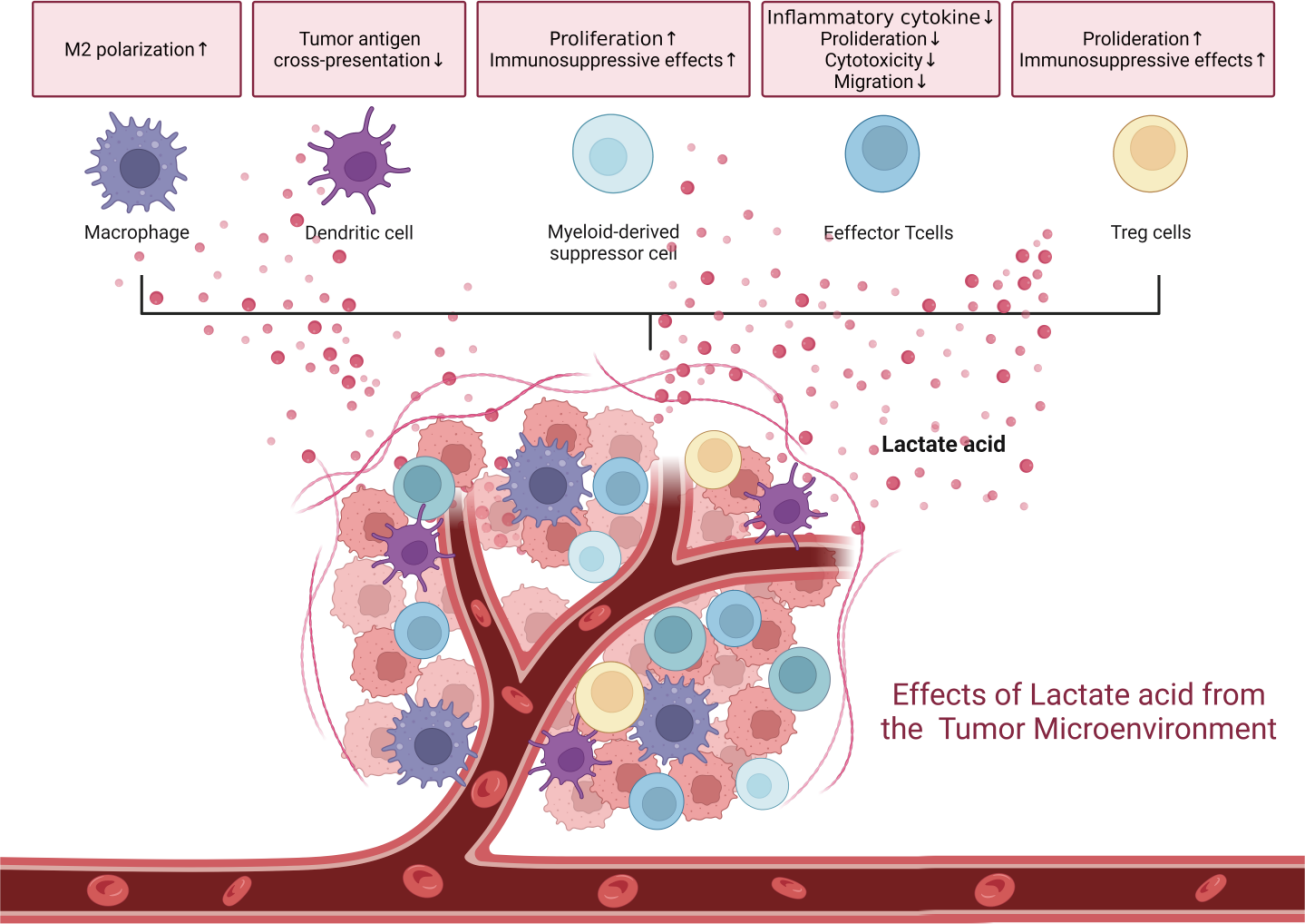
The tumor microenvironment is a hyperlactic and hypoxic milieu due to tumor proliferation. These conditions are inherently immune-suppressive, affecting various immune cells.

Lactate exists in two protonated forms, lactic acid at low pH and sodium lactate at high pH (74). Under physiological conditions, the majority of lactic acid has a negative charge. In the TME, which is typically hyperlactic with a pH ranging from 6.0-6.5, lactate exists in an acidic form. The hyperlactic milieu impacts immune cell behavior by increasing the regulatory function of myeloid-derived suppressor cells, inhibiting monocyte differentiation into dendritic cells (DCs), and compromising their antigen-presenting ability (**Figure 2**) (75–77). Tumor-associated DCs can be induced by high lactic acid in tumors, either alone or in combination with cytokines (77). Although tumor-associated macrophages (TAMs) exhibit different transcriptional and metabolic characteristics, they all have a high lactate intake. Lactate provides a supplementary carbon source for MHC-II^low^ TAM but impairs the metabolic activity of MHC-II^high^ TAM (78).

T cells detect extracellular lactate and regulate intracellular signals to maintain cell activity and homeostasis. The low pH of the TME caused by high lactic acid can lead to anergy in activated T cells and NK cells, characterized by decreased glycolysis and lactate efflux, cytotoxicity, and cytokine production (29, 32). Lactate interferes with the autophagic pathway. Naïve T cells are prone to undergo apoptosis due to a selective loss of focal adhesion kinase (FAK) family–interacting protein of 200 kDa (FIP200). Lactate suppresses FIP200 and light chain 3-II expression in naïve T cells to promote T cell apoptosis (79). In addition, lactate drives T-cell death by hindering p38 signaling protein phosphorylation and subsequently inhibits the production of IFN-γ, tumor necrosis factor α (TNF-α), and IL-2 (80).

In addition to influencing the survival of T cells, lactate affects IFN-γ production. Lactic acid instead of its sodium salt prevents CD8^+^ T and NK cells from upregulating the nuclear factor of activated T cells (NFAT), the transcription factors involved in IFN-γ transcription, leading to reduced IFN-γ production (68). Sodium lactate does not affect the IFN-γ production in CD4^+^ T cells (64). Upon the uptake of lactate, a reduced NAD^+^/NADH ratio occurs, leading to the induction and activation of NAD-dependent deacetylase SIRT1 expression (81). Then the activated SIRT1 deacetylates T-bet, a key transcription factor for Th1 differentiation, leading to its breakdown, thereby impeding Th1 differentiation (82).

The reduced NAD^+^/NADH also affects other NAD^+^-dependent enzymes including GAPDH and 3-phosphoglycerate dehydrogenase, which not only affects glycolysis but also decreases the glycolytic intermediates such as phosphoenolpyruvate and serine which are necessary for T cell proliferation (83). Leukemia cell-derived lactate interferes with T cell function and proliferation by lowering intracellular pH, which reduces transcription of glycolysis-related enzymes in CD8^+^ T cells from acute myeloid leukemia patients who relapsed after hematopoietic stem cell transplant (84).

But the impact of lactate on CD8^+^ T cells in the TME has been a subject of debate. Recently, Qiang Feng et al. found that lactate enhances the stemness of CD8^+^ T cells in tumors, leading to reduced tumor growth and improved antitumor activity when administered to tumor-bearing mice (85). This inconsistency may be due to the use of two different types of lactate: lactic acid and sodium lactate, which have distinct properties. The presence of hydrogen ions in the TME consistently affects the dynamics of lactate and sodium lactate, leading to potential inconsistencies in many studies. A recent study found that CD8^+^ T cells exhibit elevated levels of granzyme B and IFN-γ in response to a neutral sodium lactate environment; however, acidic lactate at the same concentration was found to be detrimental to CD8^+^ T cell survival (preprint, DOI: 10.1101/2021.12.14.472728). Similar to this research, sodium lactate increased the number of cytokines produced by T cells (such as IFN-γ, IL-2, and TNF-α), which slowed tumor development (86). Of note, either sodium lactate or lactic acid always benefits Treg cells. High levels of Foxp3 expression drive the metabolic flux of Treg cells, making them more tolerant to a low-sugar, high-lactate condition by downregulating c-Myc and glycolysis, improving OXPHOS and increasing NAD^+^ oxidation (32). High glucose levels impair Treg cell function and stability, whereas lactate influx and intracellular production, regulated by MCT1 (SLC16A1), are essential for maintaining Treg cell inhibitory activity (29).

**The inflammatory disease microenvironment is another hyperlactic milieu, with the most inflammatory sites displaying hypoxia.** Lactate functions through protein binding and stabilizes prolonged hypoxia (24 hours under 1% O_2_), independent of the classical regulator HIF-1α. In normoxia, the N-myc downstream-regulated gene 3 (NDRG3) is degraded *via* PHD2/VHL-dependent manner, but NDRG3 prevents degradation by binding to lactate, thereby activating the Raf-ERK pathway and promoting angiogenesis and cell growth (87). The mitochondrial antiviral-signaling protein (MAVS) functions as a lactate sensor. Direct binding of lactate to MAVS results in its inactivation, which then limits the activation of the RIG-I-like receptor (RLR) signaling pathway. Consequently, there is a decrease in the production of type I interferon in T cells, leading to prolonged inflammation and impaired T cell function (88). Inflammatory areas are characterized by increased lactate transporter activity in T cells, which results in elevated cytokine production but impairs migratory capacity (64, 89). Exposure to sodium lactate decreases glucose flux and reduces the expression of several glycolytic enzymes in CD4^+^ T cells, impairing their ability to leave inflamed tissue (64). In rheumatoid arthritis, CD4^+^ T cells exhibit compromised glycolysis, reduced levels of ROS, energy deprivation, and autophagy deficiency (26). CD4^+^ T cells express the lactate transporter SLC5A12, which is responsible for lactate sensing. SLC5A12-mediated lactate uptake stimulates IL-17 production through the activation of the nuclear PKM2/STAT3 pathway and increases fatty acid synthesis in CD4^+^ T cells; inhibition of SLC5A12 reduces lactate uptake and improves T cell function, offering a potential therapeutic strategy for the treatment of arthritis (89).

### 3.3 Lactate mediates regulatory function in a receptor-dependent manner

Lactate is a natural ligand for GPR81, activating the receptor (73). By activating AMP-activated protein kinase (AMPK) and large tumor suppressor kinases, lactate-GPR81 signaling in macrophages suppresses YAP and NF-κB subunit p65 translocation, interaction, and activation (90). This lactate-GPR81 signaling pathway in macrophages also downregulates the NOD-like receptor protein 3 (NLRP3) inflammasome by interfering with caspase 1 and IL-1β release, thereby alleviating conditions such as immune hepatitis, acute pancreatitis, and acute liver injury (91). Notably, lactate exerts its suppressive effect through GPR81 at physiologic concentrations of 3-4 mM, while high, sustained levels of lactate can lead to intracellular acidosis and enhance NLRP3-mediated responses (91). Activating the GPR81-lactate axis in antigen-presenting DCs reduces the MHC II expression and decreases the production of cAMP, IL-6, and IL-12, impairing tumor antigen presentation (92). The GPR81-lactate axis in colony macrophages and DCs is crucial for maintaining gut homeostasis and preventing colitis (93). Defective GPR81 weakens lactate signaling and increases MCT expression, suggesting a complex interplay between lactate-dependent and -independent pathways (73). Lactate is the ligand of G-protein-coupled receptor 132 (GPR132) as well. Lactate-GPR132 interaction induces protumoral M2 phenotype to facilitate tumor survival (94, 95). In high-fat diet-fed mice, activation of GPR132 by lactate in macrophages reduces proinflammatory M1 polarization and improves glucose homeostasis in adipocytes *via* the cAMP/PKA/AMPK signaling pathway (96). As a ligand for GPR81 and GPR132, lactate could regulate various signaling pathways in macrophages and affect immune responses, tumor survival, and glucose homeostasis.

## 4 Lactate regulates gene expression by affecting post-translational modifications through covalent modifications

**Figure 3:**
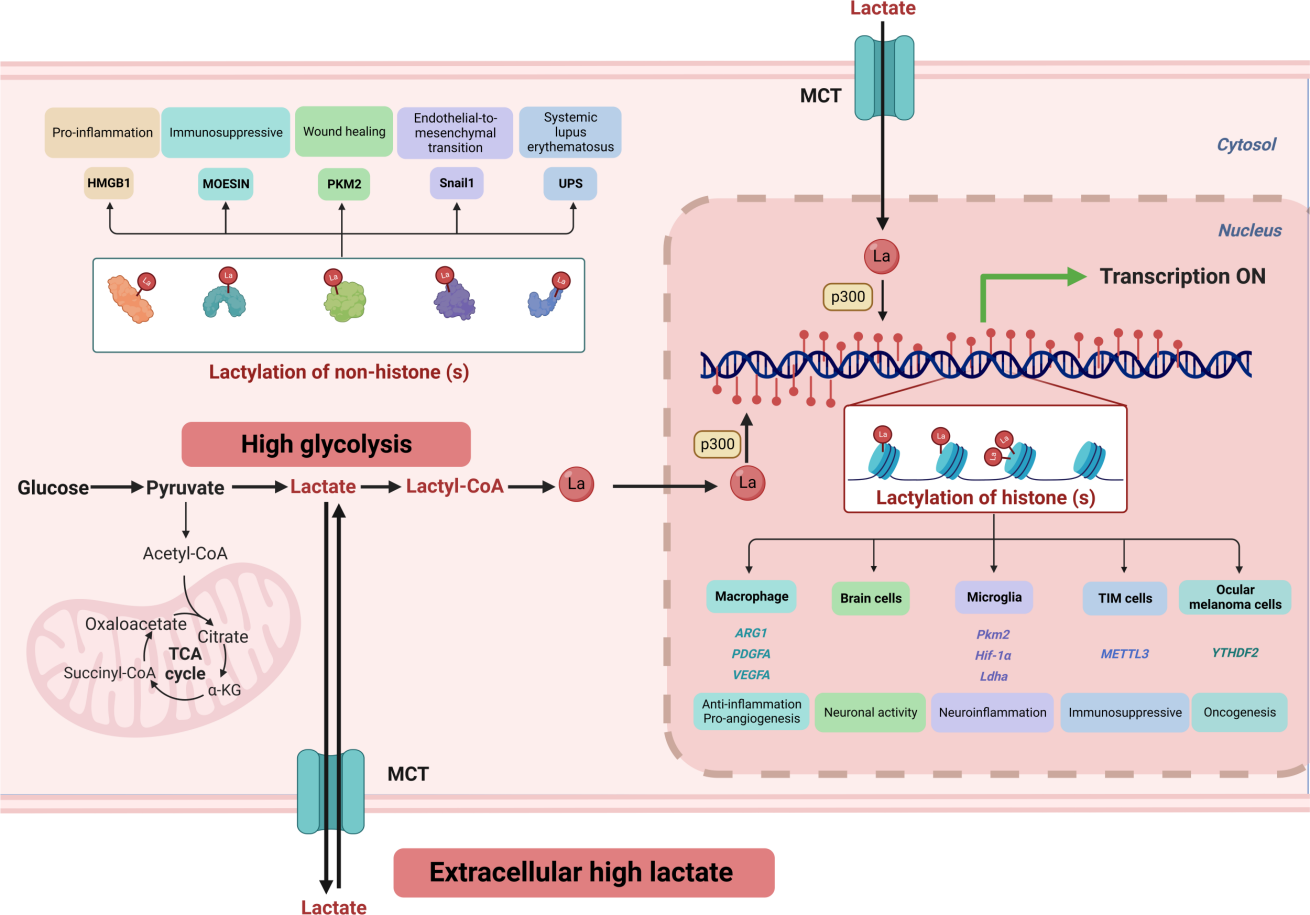
High glycolysis and an extracellular high lactate environment result in increased lactate flux. Lactate serves as a crucial substrate for covalent modification of both histone and non-histone lysine residues, playing a vital role in regulating cellular signaling and gene expression. ARG1, Arginase 1; α-KG, α-Ketoglutarate; Hif-1α, hypoxia-induced factor 1 α; HMGB1, high mobility group protein B1; Ldha, lactate dehydrogenase A; MCT, monocarboxylate transporter 1; METTL3, methyltransferase-like 3; PDGFA, platelet-derived growth factor subunit A; PKM2, pyruvate kinase M2; TCA, tricarboxylic acid cycle; TIM, Tumor-infiltrating myeloid; UPS, ubiquitin-proteasome system; VEGFA, vascular endothelial growth factor-A; YTHDF2, YTH N6-methyladenosine RNA-binding protein 2.

The modifications of nucleotide or amino acid residues in DNA and histones can reveal distinct features of the genome, affecting DNA replication, transcription, and repair (97). These modifications include acetylation, methylation, phosphorylation, ubiquitination, acylation, hydroxylation, glycosylation, and others. The histones can be modified on the free N-terminal tails or the globular structures attached to DNA, either enzymatically or non-enzymatically (98). The substrate for acylated modifications often comes from cellular metabolites, such as acetyl-CoA for acetylation. For metabolic intermediates and chromatin changes to interact, enzyme Km/Kd values and substrate concentrations must be comparable (99). Intriguingly, Yingming Zhao’s research team discovers that lactate can modify histone lactylation to regulate gene expression. Lactylation is a post-translational protein modification that involves the addition of lactic acid residue on the lysine of proteins. Histone lactylation has been identified at 26 sites in HeLa cells and 16 sites in bone marrow-derived macrophages (2). Cellular metabolic reprogramming induces an imbalance between glycolysis and the TCA cycle, leading to an increase in histone lactylation. **Figure 3** shows that lactate affects cellular activity *via* histones and non-histone lysine lactylation.

### 4.1 Lactate modifies histones and regulates epigenetics

In a study led by Yingming Zhao, lipopolysaccharide-activated M1 macrophages were found to enhance glucose metabolism and increase intracellular lactate levels, which led to histone modification and upregulation of genes involved in wound healing, such as Arginase 1 (Arg1) (2). This transformation results in the macrophages acquiring a repair phenotype, known as M2. The findings highlight the unique role of the Warburg effect in affecting epigenetics. Previous research has highlighted the involvement of B-cell adapter (BCAP) in the role of macrophages in intestinal inflammation and tissue repair. Recent discoveries indicate that the decreased production of lactate due to the absence of BCAP leads to reduced histone lactylation, resulting in diminished expression of tissue repair genes and hindering the transition of macrophages to a repair phenotype (100). During early myocardial infarction, monocytes undergo metabolic reprogramming that regulates their anti-inflammatory and pro-angiogenic functions through glycolysis and MCT-mediated histone lactylation (101). These findings underscore the critical function of lactate generation, histone lactylation, and downstream genes in negative feedback regulation, as well as cellular activity and homeostasis maintenance.

Lactate plays important roles in the brain at the molecular and behavioral levels, acting as a vital component in the neuron-astrocyte shuttle for maintaining neuronal activity. Furthermore, lactylation of lysine, which occurs in various brain cells and is regulated by neural excitation and stress, is a widespread phenomenon (102). In Alzheimer’s disease (AD), microglia exhibit pro-inflammatory signaling, accompanied by a metabolic shift from OXPHOS to glycolysis. This metabolic change results in lactate-induced modification of the H4K12 site through a positive feedback loop of glycolysis/epigenetics (H4K12-lactylation)/glycolysis (pyruvate kinase), promoting glycolytic activity and exacerbating microglia dysfunction in AD (103). Through these investigations, further insight into the involvement of lactylation in neuropsychiatric disorders has been gained.

Lactate in the environment can affect lactylation of nearby cells. Environmental lactate can impact nearby cells by promoting lactylation. Metabolic reprogramming of pulmonary myofibroblasts leads to increased glycolysis, intensifying their fibrotic phenotype. Lactate does induce the expression of fibrosis-promoting genes (ARG1, PDGFA, THBS1, and VEGFA) in macrophages through lactylation. Moreover, the fibrotic phenotype is attenuated when the lactylation writer p300 is downregulated (104). Tumor-infiltrating myeloid (TIM) cells promote an immunosuppressive environment in a TME with high lactate levels. This process is facilitated by the primary mediator of m6A alteration, methyltransferase-like 3 (METTL3), which has pro-oncogenic effects in malignancies. Lactate increases *METTL3* transcription in TIMs by altering histone H3K18 lactylation and directly enhances the catalytic activity of post-translational METTL3 protein (105). In addition, Histone H3K18 lactylation was discovered to increase the transcription of *YTHDF2*, an m6A recognition protein, which then favors the degradation of m6A-modified *PER1* and *TP53* mRNA, speeding up carcinogenesis in ocular melanoma (106). The flexibility of inflammatory Th17 and anti-inflammatory Treg cells to switch under specific circumstances has been discovered, with induction *in vitro* requiring transforming growth factor (TGF)-β. Previous research has focused on identifying the cytokine combinations responsible for this phenotypic flip, and the underlying mechanism has been debated. Recently, it was demonstrated that lactate inhibits IL-17A production by Th17 cells, while the release of IL-2 by ROS increases Foxp3 expression, leading to elevated lactylation on H3K18 (107). This modification’s involvement in epigenetic alterations appears crucial for T cell phenotypic transition. The identification of histone lactylation has introduced a new level of intricacy in our comprehension of gene expression regulation and chromatin structure.

### 4.2 Lactate modifies non-histone proteins and regulates protein activity

Lactylation of non-histone proteins has emerged as a pivotal regulatory mechanism across diverse biological processes. Perturbations in lactylation have been identified as pathological factors in many diseases.

During normal multipotent stem cell differentiation, there is a metabolic shift from glycolysis to OXPHOS. This metabolic shift is accompanied by a decrease in lactate levels, which leads to a reduction in lactylation modifications on lysine residues of the ubiquitin-proteasome system (UPS). The reduction in lactylation promotes the assembly of UPS, thereby enhancing its chymotrypsin-like activity. Impaired clearance of mitochondria during mammalian erythropoiesis is one of the mechanisms involved in the development of systemic lupus erythematosus (SLE). This disruption in the clearance process by UPS results in the accumulation of red blood cells (RBCs) containing mitochondria (Mito^+^ RBCs). These Mito^+^ RBCs are then phagocytosed by macrophages, which triggers the production of type I interferon (IFN) by macrophages (108). The TGF-β/Smad2 pathway, which mediates endothelial-to-mesenchymal transition, plays a critical role in cardiac fibrosis, and upregulation of this pathway exacerbates fibrosis. Lactylation of Snail1, a transcription factor in the TGF-β pathway, promotes its nuclear localization and enhances its ability to bind to the TGF-β promoter, resulting in increased TGF-β production (109). Lactylation of the K62 site on pyruvate kinase M2 (PKM2) improves kinase activity and helps transform pro-inflammatory macrophages into reparative phenotypes by decreasing PKM2 nuclear translocation and tetramer-to-dimer transition (110).

Treg cells can effectively function in high-lactate environments due to their strong gluconeogenesis and utilization of lactate as a carbon source. Recent research has revealed that lactylated MOESIN at position 72 could increase its affinity for TGF-β receptor I and downstream signaling pathways. This results in elevated levels of TGF-β induced Foxp3 expression and Treg cell amount (111). During sepsis, extracellular lactate is taken up by macrophages *via* MCT, leading to lactylation of the nuclear protein high mobility group box 1 (HMGB1). This results in the release of HMGB1 from macrophages as exosomes, which increases endothelial permeability due to the protein’s pro-inflammatory properties (112).

Lactylation was identified as a key global regulator in hepatocellular carcinoma (HCC), affecting 9256 non-histone protein sites (113). Interestingly, lactylation preferentially affects enzymes involved in metabolic pathways. Lactylation of K28 in adenylate kinase 2 leads to the inhibition of enzyme function and promotes HCC proliferation and metastasis. Investigating the underlying mechanisms of lactylation’s effects on protein function and its involvement in human disease will be essential to fully comprehending the role of this modification in cellular biology. Further studies are needed to shed light on these aspects.

### 4.3 Glycolytic Flux and acylation

Short-chain acyl-group-containing molecules can undergo acylation, catalyzed by histone acetyltransferases such as p300 and lysine acetyltransferase 2A (KAT2A, also known as GCN5). Lysine residues of histones can be significantly modified by certain acyl-CoA metabolites, including succinyl-CoA and malonyl-CoA. Succinyl-CoA is produced locally in the nucleus by α-ketoglutarate (α-KG) dehydrogenase, which can then be utilized by KAT2A for histone modification (114). Histone crotonylation (Kcr) results from the modification of histones by the histone acetyltransferase p300 with crotonyl-CoA, which is derived from the crotonate (a short-chain fatty acid) (115). Ketone bodies such as butyrate and β-OHB induce the production of histone lysine butyrylation (Kbu) (116) and β-hydroxybutyrylation (Kbhb) (117), respectively. Kbu and Kcr compete with acetylation for histone-binding sites (116, 118).

Changes in glycolytic flux impact the extent of histone pan-acetylation and show variation at specific modification sites. Notably, certain residues demonstrate no significant histone acetylation changes upon shifts in glycolytic flux, while approximately 40% of acetylation sites display quantitatively altered levels in response to changes in glycolysis. These modified sites exhibit a correlation with both acetyl-CoA levels and acetyl-CoA/CoA ratios (119). All histone propionylation (Kpr), butyrylation (Kbu) and 2-hydroxyisobutyrylation (Khib) demonstrated quantifiable changes in response to shifts in glycolytic flux, across all residue levels (119). Variations in glycolytic flux can modify the extent of histone acylation modifications, with the fluctuating level of glycolytic metabolites exerting a significant impact. Modifications near the globular structural domains of histones are more resistant to changes in glycolysis, while modifications in the histone tails appear to be more sensitive. These observations imply that the proximity to the metabolic environment may influence the viability of specific histone marks (119). Given the interdependent nature of lysine acylation, metabolic pathways, and their products, the kinetics of lactylation and acetylation can both show variable changes in response to glycolytic flux. Furthermore, these modifications may undergo alterations in either the same or opposite directions (2). Lactylation at specific sites serves as a marker for active promoters and exhibits a strong correlation with additional active modifications, including H3K27ac and H3K4me3 (120). Various acylations can elicit synergistic effects on non-histone proteins. For example, the lactylation and acetylation of HMGB1 synergistically increase endothelial permeability and the modified HMGB1 is released from macrophages *via* exosomes (112).

Efficient allocation of limited resources is essential for organisms to optimize growth, reproduction, and survival in their respective environments, with metabolism serving as a fundamental biological function underlying these processes. In 1958, Otto Heinrich Warburg pointed out that the switch to glycolysis was not only characteristic of tumor cell growth but also a defining feature of immune cell activation (121). Despite this insight, it was not until 2011 that the concept of immunometabolism emerged, linking the two disciplines of immunology and metabolism (122). Metabolic adaptation is now recognized as a critical initial step in immune responses, where immune cells switch between different metabolic modes based on environmental signals to perform their functions. Research on metabolic reprogramming has evolved from studying the Warburg effect to investigating fundamental energy substrates such as glucose, fatty acids, and amino acids. These substrates serve as both cofactors and substrates for epigenome-modifying enzymes, creating a link between metabolic and chromatin states. The interplay between metabolism, epigenetics, and post-transcriptional regulation is a promising yet underexplored area of research with potential to uncover mechanisms of cellular life processes.

## Author contributions

HW wrote the manuscript and draw the illustrations. HW and YZ contributed to the literature search. HH and YZ designed and supervised the study. All authors contributed to the article and approved the submitted version.

## References

[1] Nguyen HD Chatterjee S Haarberg KM Wu Y Bastian D Heinrichs J. Metabolic reprogramming of alloantigen-activated T cells after hematopoietic cell transplantation. J Clin Invest (2016) 126:1337–52. doi: 10.1172/JCI82587 PMC4811142 26950421

[2] Zhang D Tang Z Huang H Zhou G Cui C Weng Y. Metabolic regulation of gene expression by histone lactylation. Nature (2019) 574:575–80. doi: 10.1038/s41586-019-1678-1 PMC6818755 31645732

[3] Vander Heiden MG Cantley LC and Thompson CB. Understanding the warburg effect: the metabolic requirements of cell proliferation. Science (2009) 324:1029–33. doi: 10.1126/science.1160809 PMC2849637 19460998

[4] Koppenol WH Bounds PL and Dang CV. Otto Warburg's contributions to current concepts of cancer metabolism. Nat Rev Cancer (2011) 11:325–37. doi: 10.1038/nrc3038 21508971

[5] Wang R Dillon CP Shi LZ Milasta S Carter R Finkelstein D. The transcription factor myc controls metabolic reprogramming upon T lymphocyte activation. Immunity (2011) 35:871–82. doi: 10.1016/j.immuni.2011.09.021 PMC3248798 22195744

[6] Gatza E Wahl DR Opipari AW Sundberg TB Reddy P Liu C. Manipulating the bioenergetics of alloreactive T cells causes their selective apoptosis and arrests graft-versus-host disease. Sci Transl Med (2011) 3:67ra68. doi: 10.1126/scitranslmed.3001975 PMC3364290 21270339

[7] Wahl DR Byersdorfer CA Ferrara JL Opipari AW Jr. Glick GD. Distinct metabolic programs in activated T cells: opportunities for selective immunomodulation. . Immunol Rev (2012) 249:104–15. doi: 10.1111/j.1600-065X.2012.01148.x PMC3422770 22889218

[8] Nakazawa MS Keith B and Simon MC. Oxygen availability and metabolic adaptations. Nat Rev Cancer (2016) 16:663–73. doi: 10.1038/nrc.2016.84 PMC5546320 27658636

[9] O'Neill LA Kishton RJ Rathmell J. A guide to immunometabolism for immunologists. Nat Rev Immunol (2016) 16:553–65. doi: 10.1038/nri.2016.70 PMC5001910 27396447

[10] Dimeloe S Mehling M Frick C Loeliger J Bantug GR Sauder U. The immune-metabolic basis of effector memory CD4+ T cell function under hypoxic conditions. J Immunol (2016) 196:106–14. doi: 10.4049/jimmunol.1501766 26621861

[11] Delgoffe GM Kole TP Zheng Y Zarek PE Matthews KL Xiao B. The mTOR kinase differentially regulates effector and regulatory T cell lineage commitment. Immunity (2009) 30:832–44. doi: 10.1016/j.immuni.2009.04.014 PMC2768135 19538929

[12] Macintyre AN Gerriets VA Nichols AG Michalek RD Rudolph MC Deoliveira D. The glucose transporter Glut1 is selectively essential for CD4 T cell activation and effector function. Cell Metab (2014) 20:61–72. doi: 10.1016/j.cmet.2014.05.004 24930970 PMC4079750

[13] Dang EV Barbi J Yang HY Jinasena D Yu H Zheng Y. Control of T(H)17/T(reg) balance by hypoxia-inducible factor 1. Cell (2011) 146:772–84. doi: 10.1016/j.cell.2011.07.033 PMC3387678 21871655

[14] McGettrick AF O'Neill LAJ. The role of HIF in immunity and inflammation. Cell Metab (2020) 32:524–36. doi: 10.1016/j.cmet.2020.08.002 32853548

[15] Phan AT Doedens AL Palazon A Tyrakis PA Cheung KP Johnson RS and Goldrath AW. Constitutive glycolytic metabolism supports CD8(+) T cell effector memory differentiation during viral infection. Immunity (2016) 45:1024–37. doi: 10.1016/j.immuni.2016.10.017 PMC5130099 27836431

[16] Yao Y Wang L Zhou J and Zhang X. HIF-1α inhibitor echinomycin reduces acute graft-versus-host disease and preserves graft-versus-leukemia effect. J Trans Med (2017) 15:28. doi: 10.1186/s12967-017-1132-9 PMC5301444 28183349

[17] Gerriets VA Kishton RJ Johnson MO Cohen S Siska PJ Nichols AG. Foxp3 and toll-like receptor signaling balance t(reg) cell anabolic metabolism for suppression. Nat Immunol (2016) 17:1459–66. doi: 10.1038/ni.3577 PMC5215903 27695003

[18] Wang S Charbonnier LM Noval Rivas M Georgiev P Li N Gerber G. MyD88 adaptor-dependent microbial sensing by regulatory T cells promotes mucosal tolerance and enforces commensalism. Immunity (2015) 43:289–303. doi: 10.1016/j.immuni.2015.06.014 26231118 PMC4545404

[19] Cham CM Gajewski TF. Glucose availability regulates IFN-gamma production and p70S6 kinase activation in CD8+ effector T cells. J Immunol (2005) 174:4670–7. doi: 10.4049/jimmunol.174.8.4670 15814691

[20] Peng M Yin N Chhangawala S Xu K Leslie CS and Li MO. Aerobic glycolysis promotes T helper 1 cell differentiation through an epigenetic mechanism. Science (2016) 354:481–4. doi: 10.1126/science.aaf6284 PMC5539971 27708054

[21] Chang CH Curtis JD Maggi LB Jr. Faubert B Villarino AV O'Sullivan D. Posttranscriptional control of T cell effector function by aerobic glycolysis. Cell (2013) 153:1239–51. doi: 10.1016/j.cell.2013.05.016 PMC3804311 23746840

[22] Ostroukhova M Goplen N Karim MZ Michalec L Guo L Liang Q. He role of low-level lactate production in airway inflammation in asthma. Am J Physiol Lung Cell Mol Physiol (2012) 302:L300–307. doi: 10.1152/ajplung.00221.2011 PMC3289274 22080752

[23] Palmer CS Ostrowski M Gouillou M Tsai L Yu D Zhou J. Increased glucose metabolic activity is associated with CD4+ T-cell activation and depletion during chronic HIV infection. Aids (2014) 28:297–309. doi: 10.1097/QAD.0000000000000128 24335483 PMC4293200

[24] Yin Y Choi SC Xu Z Perry DJ Seay H Croker BP. Normalization of CD4+ T cell metabolism reverses lupus. . Sci Transl Med (2015) 7:274ra218. doi: 10.1126/scitranslmed.aaa0835 PMC5292723 25673763

[25] Gerriets VA Kishton RJ Nichols AG Macintyre AN Inoue M Ilkayeva O. Metabolic programming and PDHK1 control CD4+ T cell subsets and inflammation. J Clin Invest (2015) 125:194–207. doi: 10.1172/JCI76012 25437876 PMC4382238

[26] Yang Z Fujii H Mohan SV Goronzy JJ and Weyand CM. Phosphofructokinase deficiency impairs ATP generation, autophagy, and redox balance in rheumatoid arthritis T cells. J Exp Med (2013) 210:2119–34. doi: 10.1084/jem.20130252 PMC3782046 24043759

[27] Fox CJ Hammerman PS Thompson CB. Fuel feeds function: energy metabolism and the T-cell response. Nat Rev Immunol (2005) 5:844–52. doi: 10.1038/nri1710 16239903

[28] Shi LZ Wang R Huang G Vogel P Neale G Green DR and Chi H. HIF1alpha-dependent glycolytic pathway orchestrates a metabolic checkpoint for the differentiation of TH17 and treg cells. J Exp Med (2011) 208:1367–76. doi: 10.1084/jem.20110278 PMC3135370 21708926

[29] Watson MJ Vignali PDA Mullett SJ Overacre-Delgoffe AE Peralta RM Grebinoski S. Metabolic support of tumour-infiltrating regulatory T cells by lactic acid. Nature (2021) 591:645–51. doi: 10.1038/s41586-020-03045-2 PMC7990682 33589820

[30] De Rosa V Galgani M Porcellini A Colamatteo A Santopaolo M Zuchegna C. Glycolysis controls the induction of human regulatory T cells by modulating the expression of FOXP3 exon 2 splicing variants. Nat Immunol (2015) 16:1174–84. doi: 10.1038/ni.3269 PMC4868085 26414764

[31] Pacella I Procaccini C Focaccetti C Miacci S Timperi E Faicchia D. Fatty acid metabolism complements glycolysis in the selective regulatory T cell expansion during tumor growth. Proc Natl Acad Sci (2018) 115:E6546–55. doi: 10.1073/pnas.1720113115 PMC6048537 29941600

[32] Angelin A Gil-de-Gómez L Dahiya S Jiao J Guo L Levine MH. Foxp3 reprograms T cell metabolism to function in low-glucose, high-lactate environments. Cell Metab (2017) 25:1282–1293.e1287. doi: 10.1016/j.cmet.2016.12.018 28416194 PMC5462872

[33] Berod L Friedrich C Nandan A Freitag J Hagemann S Harmrolfs K. *De novo* fatty acid synthesis controls the fate between regulatory T and T helper 17 cells. Nat Med (2014) 20:1327–33. doi: 10.1038/nm.3704 25282359

[34] Michalek RD Gerriets VA Jacobs SR Macintyre AN MacIver NJ Mason EF. Cutting edge: distinct glycolytic and lipid oxidative metabolic programs are essential for effector and regulatory CD4+ T cell subsets. J Immunol (2011) 186:3299–303. doi: 10.4049/jimmunol.1003613 PMC3198034 21317389

[35] Klein Geltink RI O'Sullivan D Corrado M Bremser A Buck MD Buescher JM. Mitochondrial priming by CD28. Cell (2017) 171:385–397.e311. doi: 10.1016/j.cell.2017.08.018 28919076 PMC5637396

[36] Lee J Walsh MC Hoehn KL James DE Wherry EJ and Choi Y. Regulator of fatty acid metabolism, acetyl coenzyme a carboxylase 1, controls T cell immunity. J Immunol (2014) 192:3190–9. doi: 10.4049/jimmunol.1302985 PMC3965631 24567531

[37] Lim SA Wei J Nguyen TM Shi H Su W Palacios G. Lipid signalling enforces functional specialization of t(reg) cells in tumours. Nature (2021) 591:306–11. doi: 10.1038/s41586-021-03235-6 PMC8168716 33627871

[38] Röhrig F Schulze A. The multifaceted roles of fatty acid synthesis in cancer. Nat Rev Cancer (2016) 16:732–49. doi: 10.1038/nrc.2016.89 27658529

[39] Plitas G Konopacki C Wu K Bos PD Morrow M Putintseva EV. Regulatory T cells exhibit distinct features in human breast cancer. Immunity (2016) 45:1122–34. doi: 10.1016/j.immuni.2016.10.032 PMC5134901 27851913

[40] Wang H Franco F Tsui YC Xie X Trefny MP Zappasodi R. CD36-mediated metabolic adaptation supports regulatory T cell survival and function in tumors. Nat Immunol (2020) 21:298–308. doi: 10.1038/s41590-019-0589-5 32066953 PMC7043937

[41] Pearce EL. Metabolism in T cell activation and differentiation. Curr Opin Immunol (2010) 22:314–20. doi: 10.1016/j.coi.2010.01.018 PMC4486663 20189791

[42] O’Sullivan D van der Windt Gerritje JW Huang Stanley C-C Curtis Jonathan D Chang C-H Buck Michael D. Memory CD8+ T cells use cell-intrinsic lipolysis to support the metabolic programming necessary for development. Immunity (2014) 41:75–88. doi: 10.1016/j.immuni.2014.06.005 25001241 PMC4120664

[43] Byersdorfer CA Tkachev V Opipari AW Goodell S Swanson J Sandquist S. Effector T cells require fatty acid metabolism during murine graft-versus-host disease. Blood (2013) 122:3230–7. doi: 10.1182/blood-2013-04-495515 PMC3814737 24046012

[44] Patsoukis N Bardhan K Chatterjee P Sari D Liu B Bell LN. PD-1 alters T-cell metabolic reprogramming by inhibiting glycolysis and promoting lipolysis and fatty acid oxidation. Nat Commun (2015) 6:6692. doi: 10.1038/ncomms7692 25809635 PMC4389235

[45] Johnson MO Wolf MM Madden MZ Andrejeva G Sugiura A Contreras DC. Distinct regulation of Th17 and Th1 cell differentiation by glutaminase-dependent metabolism. Cell (2018) 175:1780–1795.e1719. doi: 10.1016/j.cell.2018.10.001 30392958 PMC6361668

[46] Chang CH Qiu J O'Sullivan D Buck MD Noguchi T Curtis JD. Metabolic competition in the tumor microenvironment is a driver of cancer progression. Cell (2015) 162:1229–41. doi: 10.1016/j.cell.2015.08.016 PMC4864363 26321679

[47] Gattinoni L Zhong XS Palmer DC Ji Y Hinrichs CS Yu Z. Wnt signaling arrests effector T cell differentiation and generates CD8+ memory stem cells. Nat Med (2009) 15:808–13. doi: 10.1038/nm.1982 PMC2707501 19525962

[48] Wherry EJ Teichgräber V Becker TC Masopust D Kaech SM Antia R. Lineage relationship and protective immunity of memory CD8 T cell subsets. Nat Immunol (2003) 4:225–34. doi: 10.1038/ni889 12563257

[49] Pearce EL Walsh MC Cejas PJ Harms GM Shen H Wang LS. Enhancing CD8 T-cell memory by modulating fatty acid metabolism. Nature (2009) 460:103–7. doi: 10.1038/nature08097 PMC2803086 19494812

[50] van der Windt GJ Everts B Chang CH Curtis JD Freitas TC Amiel E. Mitochondrial respiratory capacity is a critical regulator of CD8+ T cell memory development. Immunity (2012) 36:68–78. doi: 10.1016/j.immuni.2011.12.007 22206904 PMC3269311

[51] Sukumar M Liu J Ji Y Subramanian M Crompton JG Yu Z. Inhibiting glycolytic metabolism enhances CD8+ T cell memory and antitumor function. J Clin Invest (2013) 123:4479–88. doi: 10.1172/JCI69589 PMC3784544 24091329

[52] Hegedus A Kavanagh Williamson M Huthoff H. HIV-1 pathogenicity and virion production are dependent on the metabolic phenotype of activated CD4+ T cells. Retrovirology (2014) 11:98. doi: 10.1186/s12977-014-0098-4 25421745 PMC4252996

[53] Manel N Kim FJ Kinet S Taylor N Sitbon M and Battini JL. The ubiquitous glucose transporter GLUT-1 is a receptor for HTLV. Cell (2003) 115:449–59. doi: 10.1016/S0092-8674(03)00881-X 14622599

[54] Killian MS Johnson C Teque F Fujimura S and Levy JA. Natural suppression of human immunodeficiency virus type 1 replication is mediated by transitional memory CD8+ T cells. J Virol (2011) 85:1696–705. doi: 10.1128/JVI.01120-10 PMC3028886 21147929

[55] Angin M Volant S Passaes C Lecuroux C Monceaux V Dillies MA. Metabolic plasticity of HIV-specific CD8(+) T cells is associated with enhanced antiviral potential and natural control of HIV-1 infection. Nat Metab (2019) 1:704–16. doi: 10.1038/s42255-019-0081-4 32694646

[56] Loisel-Meyer S Swainson L Craveiro M Oburoglu L Mongellaz C Costa C. Glut1-mediated glucose transport regulates HIV infection. Proc Natl Acad Sci U.S.A. (2012) 109:2549–54. doi: 10.1073/pnas.1121427109 PMC3289356 22308487

[57] Valle-Casuso JC Angin M Volant S Passaes C Monceaux V Mikhailova A. Cellular metabolism is a major determinant of HIV-1 reservoir seeding in CD4(+) T cells and offers an opportunity to tackle infection. Cell Metab (2019) 29:611–626.e615. doi: 10.1016/j.cmet.2018.11.015 30581119

[58] DeBerardinis RJ Mancuso A Daikhin E Nissim I Yudkoff M Wehrli S and Thompson CB. Beyond aerobic glycolysis: transformed cells can engage in glutamine metabolism that exceeds the requirement for protein and nucleotide synthesis. Proc Natl Acad Sci U.S.A. (2007) 104:19345–50. doi: 10.1073/pnas.0709747104 PMC2148292 18032601

[59] Dzeja PP Terzic A. Phosphotransfer networks and cellular energetics. J Exp Biol (2003) 206:2039–47. doi: 10.1242/jeb.00426 12756286

[60] Bittl JA Ingwall JS. Reaction rates of creatine kinase and ATP synthesis in the isolated rat heart. A 31P NMR magnetization transfer study. J Biol Chem (1985) 260:3512–7. doi: 10.1016/S0021-9258(19)83652-9 3972835

[61] Slavov N Botstein D. Decoupling nutrient signaling from growth rate causes aerobic glycolysis and deregulation of cell size and gene expression. Mol Biol Cell (2013) 24:157–68. doi: 10.1091/mbc.e12-09-0670 PMC3541962 23135997

[62] Locasale JW Cantley LC. Metabolic flux and the regulation of mammalian cell growth. Cell Metab (2011) 14:443–51. doi: 10.1016/j.cmet.2011.07.014 PMC3196640 21982705

[63] Haas R Cucchi D Smith J Pucino V Macdougall CE and Mauro C. Intermediates of metabolism: from bystanders to signalling molecules. Trends Biochem Sci (2016) 41:460–71. doi: 10.1016/j.tibs.2016.02.003 26935843

[64] Haas R Smith J Rocher-Ros V Nadkarni S Montero-Melendez T D'Acquisto F. Lactate regulates metabolic and pro-inflammatory circuits in control of T cell migration and effector functions. PloS Biol (2015) 13:e1002202. doi: 10.1371/journal.pbio.1002202 26181372 PMC4504715

[65] Tannahill GM Curtis AM Adamik J Palsson-McDermott EM McGettrick AF Goel G. Succinate is an inflammatory signal that induces IL-1β through HIF-1α. Nature (2013) 496:238–42. doi: 10.1038/nature11986 PMC4031686 23535595

[66] Koide S Iwata S Matsuzawa H and Ohta T. Crystallization of allosteric l-lactate dehydrogenase from thermus caldophilus and preliminary crystallographic data. J Biochem (1991) 109:6–7. 2016275

[67] Song W Li D Tao L Luo Q and Chen L. Solute carrier transporters: the metabolic gatekeepers of immune cells. Acta Pharm Sin B (2020) 10:61–78. doi: 10.1016/j.apsb.2019.12.006 31993307 PMC6977534

[68] Brand A Singer K Koehl GE Kolitzus M Schoenhammer G Thiel A. LDHA-associated lactic acid production blunts tumor immunosurveillance by T and NK cells. Cell Metab (2016) 24:657–71. doi: 10.1016/j.cmet.2016.08.011 27641098

[69] Colegio OR Chu NQ Szabo AL Chu T Rhebergen AM Jairam V. Functional polarization of tumour-associated macrophages by tumour-derived lactic acid. Nature (2014) 513:559–63. doi: 10.1038/nature13490 PMC4301845 25043024

[70] Faubert B Li KY Cai L Hensley CT Kim J Zacharias LG. Lactate metabolism in human lung tumors. Cell (2017) 171:358–371.e359. doi: 10.1016/j.cell.2017.09.019 28985563 PMC5684706

[71] Hui S Ghergurovich JM Morscher RJ Jang C Teng X Lu W. Glucose feeds the TCA cycle *via* circulating lactate. Nature (2017) 551:115–8. doi: 10.1038/nature24057 PMC5898814 29045397

[72] Liu C Wu J Zhu J Kuei C Yu J Shelton J. Lactate inhibits lipolysis in fat cells through activation of an orphan G-protein-coupled receptor, GPR81. J Biol Chem (2009) 284:2811–22. doi: 10.1074/jbc.M806409200 19047060

[73] Roland CL Arumugam T Deng D Liu SH Philip B Gomez S. Cell surface lactate receptor GPR81 is crucial for cancer cell survival. Cancer Res (2014) 74:5301–10. doi: 10.1158/0008-5472.CAN-14-0319 PMC4167222 24928781

[74] Robergs RA Ghiasvand F and Parker D. Biochemistry of exercise-induced metabolic acidosis. Am J Physiol Regul Integr Comp Physiol (2004) 287:R502–516. doi: 10.1152/ajpregu.00114.2004 15308499

[75] Husain Z Seth P and Sukhatme VP. Tumor-derived lactate and myeloid-derived suppressor cells: linking metabolism to cancer immunology. Oncoimmunology (2013) 2:e26383. doi: 10.4161/onci.26383 24404426 PMC3881600

[76] Puig-Kröger A Pello OM Muñiz-Pello O Selgas R Criado G Bajo MA. Peritoneal dialysis solutions inhibit the differentiation and maturation of human monocyte-derived dendritic cells: effect of lactate and glucose-degradation products. J Leukoc Biol (2003) 73:482–92. doi: 10.1189/jlb.0902451 12660223

[77] Gottfried E Kunz-Schughart LA Ebner S Mueller-Klieser W Hoves S Andreesen R. Tumor-derived lactic acid modulates dendritic cell activation and antigen expression. Blood (2006) 107:2013–21. doi: 10.1182/blood-2005-05-1795 16278308

[78] Geeraerts X Fernández-Garcia J Hartmann FJ de Goede KE Martens L Elkrim Y. Macrophages are metabolically heterogeneous within the tumor microenvironment. Cell Rep (2021) 37:110171. doi: 10.1016/j.celrep.2021.110171 34965415

[79] Xia H Wang W Crespo J Kryczek I Li W Wei S. Suppression of FIP200 and autophagy by tumor-derived lactate promotes naïve T cell apoptosis and affects tumor immunity. Sci Immunol (2017) 2(17):eaan4631. doi: 10.1126/sciimmunol.aan4631 29150439 PMC5774333

[80] Mendler AN Hu B Prinz PU Kreutz M Gottfried E and Noessner E. Tumor lactic acidosis suppresses CTL function by inhibition of p38 and JNK/c-jun activation. Int J Cancer (2012) 131:633–40. doi: 10.1002/ijc.26410 21898391

[81] Gambini J Gomez-Cabrera MC Borras C Valles SL Lopez-Grueso R Martinez-Bello VE. Free [NADH]/[NAD(+)] regulates sirtuin expression. Arch Biochem Biophys (2011) 512:24–9. doi: 10.1016/j.abb.2011.04.020 21575591

[82] Comito G Iscaro A Bacci M Morandi A Ippolito L Parri M. Lactate modulates CD4+ T-cell polarization and induces an immunosuppressive environment, which sustains prostate carcinoma progression *via* TLR8/miR21 axis. Oncogene (2019) 38:3681–95. doi: 10.1038/s41388-019-0688-7 30664688

[83] Quinn WJ 3rd Jiao J TeSlaa T Stadanlick J Wang Z Wang L. Lactate limits T cell proliferation *via* the NAD(H) redox state. Cell Rep (2020) 33:108500. doi: 10.1016/j.celrep.2020.108500 33326785 PMC7830708

[84] Uhl FM Chen S O'Sullivan D Edwards-Hicks J Richter G Haring E. Metabolic reprogramming of donor T cells enhances graft-versus-leukemia effects in mice and humans. Sci Transl Med (2020) 12(567):eabb8969. doi: 10.1126/scitranslmed.abb8969 33115954 PMC8529950

[85] Feng Q Liu Z Yu X Huang T Chen J Wang J. Lactate increases stemness of CD8 + T cells to augment anti-tumor immunity. Nat Commun (2022) 13:4981. doi: 10.1038/s41467-022-32521-8 36068198 PMC9448806

[86] Wen J Cheng S Zhang Y Wang R Xu J Ling Z. Lactate anions participate in T cell cytokine production and function. Sci China Life Sci (2021) 64:1895–905. doi: 10.1007/s11427-020-1887-7 33580429

[87] Lee DC Sohn HA Park ZY Oh S Kang YK Lee KM. A lactate-induced response to hypoxia. Cell (2015) 161:595–609. doi: 10.1016/j.cell.2015.03.011 25892225

[88] Zhang W Wang G Xu ZG Tu H Hu F Dai J. Lactate is a natural suppressor of RLR signaling by targeting MAVS. Cell (2019) 178:176–189.e115. doi: 10.1016/j.cell.2019.05.003 31155231 PMC6625351

[89] Pucino V Certo M Bulusu V Cucchi D Goldmann K Pontarini E. Lactate buildup at the site of chronic inflammation promotes disease by inducing CD4(+) T cell metabolic rewiring. Cell Metab (2019) 30:1055–1074.e1058. doi: 10.1016/j.cmet.2019.10.004 31708446 PMC6899510

[90] Yang K Xu J Fan M Tu F Wang X Ha T. Lactate suppresses macrophage pro-inflammatory response to LPS stimulation by inhibition of YAP and NF-κB activation *via* GPR81-mediated signaling. Front Immunol (2020) 11:587913. doi: 10.3389/fimmu.2020.587913 33123172 PMC7573489

[91] Hoque R Farooq A Ghani A Gorelick F and Mehal WZ. Lactate reduces liver and pancreatic injury in toll-like receptor- and inflammasome-mediated inflammation *via* GPR81-mediated suppression of innate immunity. Gastroenterology (2014) 146:1763–74. doi: 10.1053/j.gastro.2014.03.014 PMC4104305 24657625

[92] Brown TP Bhattacharjee P Ramachandran S Sivaprakasam S Ristic B Sikder MOF and Ganapathy V. The lactate receptor GPR81 promotes breast cancer growth *via* a paracrine mechanism involving antigen-presenting cells in the tumor microenvironment. Oncogene (2020) 39:3292–304. doi: 10.1038/s41388-020-1216-5 32071396

[93] Ranganathan P Shanmugam A Swafford D Suryawanshi A Bhattacharjee P Hussein MS. GPR81, a cell-surface receptor for lactate, regulates intestinal homeostasis and protects mice from experimental colitis. J Immunol (2018) 200:1781–9. doi: 10.4049/jimmunol.1700604 PMC5858928 29386257

[94] Chen P Zuo H Xiong H Kolar MJ Chu Q Saghatelian A. Gpr132 sensing of lactate mediates tumor-macrophage interplay to promote breast cancer metastasis. Proc Natl Acad Sci U.S.A. (2017) 114:580–5. doi: 10.1073/pnas.1614035114 PMC5255630 28049847

[95] Vadevoo SMP Gunassekaran GR Lee C Lee N Lee J Chae S. The macrophage odorant receptor Olfr78 mediates the lactate-induced M2 phenotype of tumor-associated macrophages. Proc Natl Acad Sci (2021) 118:e2102434118. doi: 10.1073/pnas.2102434118 34504016 PMC8449333

[96] Cai H Wang X Zhang Z Chen J Wang F Wang L and Liu J. Moderate l-lactate administration suppresses adipose tissue macrophage M1 polarization to alleviate obesity-associated insulin resistance. J Biol Chem (2022) 298:101768. doi: 10.1016/j.jbc.2022.101768 35218776 PMC8941214

[97] Hyun K Jeon J Park K and Kim J. Writing, erasing and reading histone lysine methylations. Exp Mol Med (2017) 49:e324–4. doi: 10.1038/emm.2017.11 PMC6130214 28450737

[98] Dai Z Ramesh V and Locasale JW. The evolving metabolic landscape of chromatin biology and epigenetics. Nat Rev Genet (2020) 21:737–53. doi: 10.1038/s41576-020-0270-8 PMC8059378 32908249

[99] Reid MA Dai Z and Locasale JW. The impact of cellular metabolism on chromatin dynamics and epigenetics. Nat Cell Biol (2017) 19:1298–306. doi: 10.1038/ncb3629 PMC5886854 29058720

[100] Irizarry-Caro RA McDaniel MM Overcast GR Jain VG Troutman TD and Pasare C. TLR signaling adapter BCAP regulates inflammatory to reparatory macrophage transition by promoting histone lactylation. Proc Natl Acad Sci U.S.A. (2020) 117:30628–38. doi: 10.1073/pnas.2009778117 PMC7720107 33199625

[101] Wang N Wang W Wang X Mang G Chen J Yan X. Histone lactylation boosts reparative gene activation post-myocardial infarction. Circ Res (2022) 131:893–908. doi: 10.1161/CIRCRESAHA.122.320488 36268709

[102] Hagihara H Shoji H Otabi H Toyoda A Katoh K Namihira M and Miyakawa T. Protein lactylation induced by neural excitation. Cell Rep (2021) 37:109820. doi: 10.1016/j.celrep.2021.109820 34644564

[103] Pan R-Y He L Zhang J Liu X Liao Y Gao J. Positive feedback regulation of microglial glucose metabolism by histone H4 lysine 12 lactylation in alzheimer’s disease. Cell Metab (2022) 34:634–648.e636. doi: 10.1016/j.cmet.2022.02.013 35303422

[104] Cui H Xie N Banerjee S Ge J Jiang D Dey T. Lung myofibroblasts promote macrophage profibrotic activity through lactate-induced histone lactylation. Am J Respir Cell Mol Biol (2021) 64:115–25. doi: 10.1165/rcmb.2020-0360OC PMC7780997 33074715

[105] Xiong J He J Zhu J Pan J Liao W Ye H. Lactylation-driven METTL3-mediated RNA m(6)A modification promotes immunosuppression of tumor-infiltrating myeloid cells. Mol Cell (2022) 82:1660–1677.e1610. doi: 10.1016/j.molcel.2022.02.033 35320754

[106] Yu J Chai P Xie M Ge S Ruan J Fan X and Jia R. Histone lactylation drives oncogenesis by facilitating m6A reader protein YTHDF2 expression in ocular melanoma. Genome Biol (2021) 22:85. doi: 10.1186/s13059-021-02308-z 33726814 PMC7962360

[107] Lopez Krol A Nehring HP Krause FF Wempe A Raifer H Nist A. Lactate induces metabolic and epigenetic reprogramming of pro-inflammatory Th17 cells . EMBO Rep (2022) 23(12):e54685. doi: 10.15252/embr.202254685 36215678 PMC9724659

[108] Caielli S Cardenas J de Jesus AA Baisch J Walters L Blanck JP. Erythroid mitochondrial retention triggers myeloid-dependent type I interferon in human SLE. Cell (2021) 184:4464–4479.e4419. doi: 10.1016/j.cell.2021.07.021 34384544 PMC8380737

[109] Fan M Yang K Wang X Chen L Gill PS Ha T. Lactate promotes endothelial-to-mesenchymal transition via Snail1 lactylation after myocardial infarction. . Sci Adv (2023) 9:eadc9465. doi: 10.1126/sciadv.adc9465 36735787 PMC9897666

[110] Wang J Yang P Yu T Gao M Liu D Zhang J. Lactylation of PKM2 suppresses inflammatory metabolic adaptation in pro-inflammatory macrophages. Int J Biol Sci (2022) 18:6210–25. doi: 10.7150/ijbs.75434 PMC9682528 36439872

[111] Gu J Zhou J Chen Q Xu X Gao J Li X. Tumor metabolite lactate promotes tumorigenesis by modulating MOESIN lactylation and enhancing TGF-β signaling in regulatory T cells. Cell Rep (2022) 39:110986. doi: 10.1016/j.celrep.2022.110986 35732125

[112] Yang K Fan M Wang X Xu J Wang Y Tu F. Lactate promotes macrophage HMGB1 lactylation, acetylation, and exosomal release in polymicrobial sepsis. Cell Death Differ (2022) 29:133–46. doi: 10.1038/s41418-021-00841-9 PMC8738735 34363018

[113] Yang Z Yan C Ma J Peng P Ren X Cai S. Lactylome analysis suggests lactylation-dependent mechanisms of metabolic adaptation in hepatocellular carcinoma. Nat Metab (2023) 5(1):61–79. doi: 10.1038/s42255-022-00710-w 36593272

[114] Wang Y Guo YR Liu K Yin Z Liu R Xia Y. KAT2A coupled with the α-KGDH complex acts as a histone H3 succinyltransferase. Nature (2017) 552:273–7. doi: 10.1038/nature25003 PMC5841452 29211711

[115] Sabari BR Tang Z Huang H Yong-Gonzalez V Molina H Kong HE. Intracellular crotonyl-CoA stimulates transcription through p300-catalyzed histone crotonylation. Mol Cell (2015) 58:203–15. doi: 10.1016/j.molcel.2015.02.029 PMC4501262 25818647

[116] Goudarzi A Zhang D Huang H Barral S Kwon OK Qi S. Dynamic competing histone H4 K5K8 acetylation and butyrylation are hallmarks of highly active gene promoters. Mol Cell (2016) 62:169–80. doi: 10.1016/j.molcel.2016.03.014 PMC4850424 27105113

[117] Xie Z Zhang D Chung D Tang Z Huang H Dai L. Metabolic regulation of gene expression by histone lysine β-hydroxybutyrylation. Mol Cell (2016) 62:194–206. doi: 10.1016/j.molcel.2016.03.036 27105115 PMC5540445

[118] Gowans GJ Bridgers JB Zhang J Dronamraju R Burnetti A King DA. Recognition of histone crotonylation by Taf14 links metabolic state to gene expression. Mol Cell (2019) 76:909–921.e903. doi: 10.1016/j.molcel.2019.09.029 31676231 PMC6931132

[119] Cluntun AA Huang H Dai L Liu X Zhao Y and Locasale JW. The rate of glycolysis quantitatively mediates specific histone acetylation sites. Cancer Metab (2015) 3:10. doi: 10.1186/s40170-015-0135-3 26401273 PMC4579576

[120] Galle E Wong CW Ghosh A Desgeorges T Melrose K Hinte LC. H3K18 lactylation marks tissue-specific active enhancers. Genome Biol (2022) 23:207. doi: 10.1186/s13059-022-02775-y 36192798 PMC9531456

[121] Warburg O Gawehn K and Geissler AW. Metabolism of leukocytes. Z Naturforsch B (1958) 13b:515–6. doi: 10.1515/znb-1958-0806 13593654

[122] Mathis D Shoelson SE. Immunometabolism: an emerging frontier. Nat Rev Immunol (2011) 11:81. doi: 10.1038/nri2922 21469396 PMC4784680

